# Perception of Salicylic Acid in *Physcomitrella patens*

**DOI:** 10.3389/fpls.2017.02145

**Published:** 2017-12-18

**Authors:** Yujun Peng, Tongjun Sun, Yuelin Zhang

**Affiliations:** Department of Botany, University of British Columbia, Vancouver, BC, Canada

**Keywords:** salicylic acid, NPR1, NPR3, NPR4, plant immunity, SA receptor

## Abstract

Salicylic acid (SA) is a key signaling molecule in plant immunity. Two types of SA receptors, NPR1 and NPR3/NPR4, were reported to be involved in the perception of SA in Arabidopsis. SA is also synthesized in the non-vascular moss *Physcomitrella patens* following pathogen infection. Sequence analysis revealed that there is only one NPR1/NPR3/NPR4-like protein in *P. patens*. This agrees with the phylogenetic study that showed the divergence of NPR1 and NPR3/NPR4 from the same ancestor during the evolution of higher plants. Intriguingly, expression of the *P. patens NPR1/NPR3/NPR4*-like gene in Arabidopsis does not complement the constitutive defense phenotype of the *npr3 npr4* double mutant, but can partially rescue the mutant phenotypes of *npr1-1*, suggesting that it functions as an NPR1-like positive regulator of SA-mediated immunity and *P. patens* does not have an SA receptor functioning similarly as NPR3/NPR4. Future characterization of the *P. patens* NPR1-like protein and analysis of its functions through knockout and biochemical approaches will help us better understand how SA is perceived and what its functions are in *P. patens*.

Systemic acquired resistance (SAR) is a plant immune response induced after primary infection, which leads to enhanced resistance to a broad spectrum of pathogens in distal tissue (Durrant and Dong, 2004). Early studies of SAR identified increased accumulation of salicylic acid (SA) and induction of *PATHOGENESIS RELATED* (*PR*) gene expression as two key features of SAR (Malamy et al., 1990; Metraux et al., 1990; Rasmussen et al., 1991; Ward et al., 1991; Yalpani et al., 1991; Uknes et al., 1992). Clear evidence showed that SA accumulation is necessary for SAR, as blocking SA accumulation by expressing a bacterial salicylate hydroxylase in transgenic plants results in low SA levels and loss of SAR (Gaffney et al., 1993). In addition, two SA-deficient mutants, *sid2* and *eds5*, display enhanced susceptibility to pathogens and compromised SAR (Nawrath and Metraux, 1999; Wildermuth et al., 2001). On the other hand, exogenous application of SA or its analogs is sufficient to induce *PR* gene expression and resistance to pathogen infection (White, 1979; Metraux et al., 1991; Gorlach et al., 1996). These studies suggest that SA is a key signaling molecule in plant immunity.

Several genetic screens conducted in Arabidopsis to identify signaling components downstream of SA led to the isolation of a large number of *npr1* alleles (Cao et al., 1994; Delaney et al., 1995; Shah et al., 1997). In *npr1* mutants, SA-induced *PR* gene expression and resistance to pathogens are blocked, suggesting that NPR1 is a key immune regulator downstream of SA. *NPR1* encodes a protein containing two protein–protein interaction motifs: a BTB/POZ (broad-complex, tram track, and bric-a-brac/poxvirus, zinc finger) domain at the N-terminus and an ankyrin-repeat domain in the middle of the protein (Cao et al., 1997; Ryals et al., 1997). NPR1 binds to SA *in vitro*, suggesting that it may function as a receptor for SA (Wu et al., 2012; Manohar et al., 2014). Yeast two-hybrid screens identified several TGA transcription factors as interactors of NPR1 (Zhang et al., 1999; Despres et al., 2000; Zhou et al., 2000). TGAs bind to the SA-responsive element (*as-1*) in the *PR1* promoter that are required for the induction of *PR* gene expression by SA, suggesting that they are important for regulating *PR1* expression (Zhang et al., 1999). Knockout analysis of TGA2/TGA5/TGA6 further revealed that induction of *PR1* expression and pathogen resistance by the SA analog INA was abolished in the *tga2 tga5 tga6* triple mutant, but unaffected in *tga6* or *tga2 tga5* mutant plants, suggesting that TGA2/TGA5/TGA6 function redundantly as positive regulators of SA-induced defense responses (Zhang et al., 2003).

Two paralogs of NPR1, NPR3 and NPR4, also interact with TGA2/TGA5/TGA6, but they function as negative regulators of *PR* gene expression and pathogen resistance (Zhang et al., 2006). The *npr3 npr4* double mutants exhibit elevated basal *PR* gene expression and enhanced resistance against pathogens. Similar to NPR1, NPR3 and NPR4 also bind to SA, suggesting that they also function as SA receptors (Fu et al., 2012). They were proposed to negatively regulate plant immunity by degrading NPR1 in response to SA (Fu et al., 2012).

*Physcomitrella patens* is the first bryophyte to have its whole genome sequenced and has been widely used in studying evolutionary changes during the evolution of land plants (Rensing et al., 2008). Few studies have been carried out on the immune system of *P. patens*. It was shown that SA levels in *P. patens* increase rapidly after inoculation with *Botrytis cinerea* (Ponce De Leon et al., 2012). Exogenous application of SA also strongly induces the expression of a *PAL* gene in *P. patens* (Ponce De Leon et al., 2012), suggesting that SA is synthesized and perceived during pathogen infection.

To better understand how SA is perceived in *P. patens*, we carried out BLAST searches to look for homologs of NPR1 in *P. patens*. Search of the NCBI database found that *Pp3c19_7560* encodes an NPR1 homolog. Search of the JGI Phytozome database revealed that another predicted protein encoded by *Pp3c21_7570* also has high similarity with Arabidopsis NPR1. To determine the actual coding sequences of these two *NPR1-like* genes in *P. patens*, we sequenced their cDNAs amplified by RT-PCR. Analysis of the cDNA sequences revealed that the predicted *Pp3c19_7560* gene model is incorrect and there is an early frame shift in the cDNA, resulting in the truncation of the encoded protein, suggesting that *Pp3c19_7560* is likely a pseudogene. The cDNA sequence of *Pp3c21_7570* is also different from the predicted mRNA sequence. The first exon is larger than predicted.

The predicted Pp3c21_7570 protein based on the cDNA sequence shows 34% identity and 53% similarity with Arabidopsis NPR1, 37% identity and 55% similarity with NPR3, and 36% identity and 56% similarity with NPR4. Similar to NPR1 and NPR3/NPR4, it contains a conserved N-terminal BTB/POZ domain, a central ankyrin-repeat domain and a C-terminal domain with nuclear localization signals (**Figure 1A**). In Arabidopsis NPR1, Cys521, and Cys529 in the C-terminal domain were reported to be required for SA-binding, but they are not universally conserved in NPR1 orthologs (Wu et al., 2012). These two residues are not conserved in Pp3c21_7570 and Arabidopsis NPR3/NPR4 either. Most likely SA-binding in SA receptors involves in additional amino acid residues. Phylogenetic analysis of proteins in the NPR1 family from different plant species revealed that NPR1 and NPR3/NPR4 diverged from Pp3c21_7570 during evolution of higher plants (**Figure 1B**). It is not obvious whether Pp3c21_7570 is more related to Arabidopsis NPR1 or NPR3/NPR4 simply from the phylogenetic tree.

**FIGURE 1:**
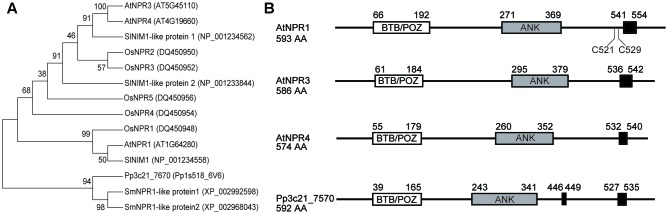
*Pp3C21_7570* encodes an NPR1-like protein in *Physcomitrella patens*. **(A)** Phylogenetic analysis of Pp3C21_7570, Arabidopsis NPR1/NPR3/NPR4 and NPR1/NPR3/NPR4-like proteins in rice (OsNPR1, OsNPR2, OsNPR3, OsNPR4, and OsNPR5), tomato (SlNIM1, SlNIM1-like protein 1, and SlNIM1-like protein 2) and the lycophyte *Selaginella moellendorffii* (SmNPR1-like protein 1 and SmNPR1-like protein 2). The protein sequences were aligned by MEGA version 7.0.26, and the maximum likelihood tree was generated using the Maximum Likelihood method. Bootstrap replication (500 replications) was used for statistical support for the nodes in the phylogenetic tree. **(B)** Predicted protein structures of Arabidopsis NPR1/NPR3/NPR4 and Pp3C21_7570. BTB/POZ domain (white box), Ankyrin-repeat (gray box), and nuclear localization signals (black box) are shown.

To determine whether Pp3c21_7570 has similar functions as NPR3/4 or NPR1, we generated a construct expressing *Pp3c21_7570* driven by the 35S promoter and transformed it into Arabidopsis *npr3-2 npr4-2* and *npr1-1* mutants to test whether it can complement the mutant phenotype of *npr3-2 npr4-2* or *npr1-1*. As shown in **Figure 2A**, the *35S::Pp3c21_7570* transgenic plants in the *npr3-2 npr4-2* double mutant background still exhibit elevated basal *PR1* expression, and the expression levels of *PR1* in the transgenic lines are actually higher than in *npr3-2 npr4-2*. Similarly, the enhanced resistance to *H.a.* Noco2 in *npr3-2 npr4-2* is unaffected in the *35S::Pp3c21_7570* transgenic lines (**Figure 2B**). These data suggest that the *35S::Pp3c21_7570* transgene does not complement the mutant phenotypes of *npr3-2 npr4-2*.

**FIGURE 2:**
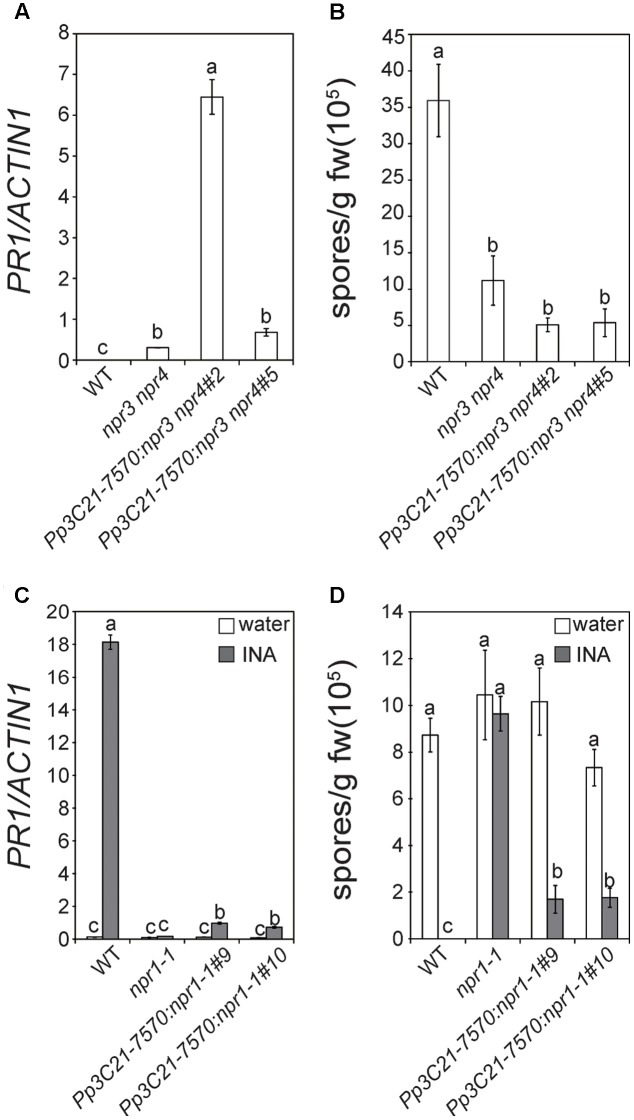
Characterization of transgenic plants expressing *Pp3C21_7570* in the Arabidopsis *npr3-2 npr4-2* and *npr1-1* mutants. **(A)** Expression of *PR1* in 2-week-old seedlings of wild type (WT), *npr3-2 npr4-2* and *35S::Pp3C21_7570* transgenic lines in *npr3-2 npr4* background (line #2 and #5). Statistical differences among the samples are labeled with different letters (*P* < 0.01, one-way ANOVA; *n* = 3). **(B)** Growth of *H.a.* Noco2 on WT, *npr3-2 npr4-2* and *35S::Pp3C21_7570* transgenic plants in *npr3-2 npr4-2* background (line #2 and #5). Two-week old seedlings were sprayed with *H.a.* Noco2 spores at a concentration of 50,000 spores/ml. Statistical differences among the samples are labeled with different letters (*P* < 0.01, one-way ANOVA; *n* = 4). **(C)** Expression level of *PR1* in WT, *npr1-1* and *35S::Pp3C21_7570* transgenic lines in *npr1-1* background (line #9 and #10) before and after INA treatment. 11-day-old seedlings were sprayed with 0.33 mM INA. Samples were collected before treatment and 48 h after treatment. Statistical differences among the samples are labeled with different letters (*P* < 0.01, one-way ANOVA; *n* = 3). **(D)** Growth of *H.a.* Noco2 on WT, *npr1-1* and *35S::Pp3C21_7570* transgenic lines in *npr1-1* background (line #9 and #10). Twelve-day-old plants were sprayed with water or 0.65 mM INA 3 days before spraying with *H.a.* Noco2 spores at a concentration of 20,000 spores/ml. Statistical differences among the samples are labeled with different letters (*P* < 0.01, one-way ANOVA; *n* = 4). The coding region of *Pp3C21_7570* was amplified by RT-PCR and cloned into a modified pCambia1300 vector with a 35S promoter. *35S::Pp3C21_7570* transgenic lines were generated by transforming Arabidopsis *npr3-2, npr4-2*, and *npr1-1* mutant plants with *Agrobacteria* carrying the construct. Expression levels of *PR1* in **(A,C)** were determined by quantitative RT-PCR. Values were normalized by the expression levels of *Actin1*. Quantitative analysis of *H.a.* Noco2 growth in **(B,D)** was carried out as previously described (Bi et al., 2010). After spraying with *H.a.* Noco2 spores, plants were grown at 18°C under 12 h day/12 h night cycle for 7 days before the conidiospores on the plants were quantified.

In the *35S::Pp3c21_7570* transgenic lines in the *npr1-1* background, INA-induced *PR1* expression is partially restored (**Figure 2C**). Similarly, INA-induced resistance to *H.a.* Noco2 is largely restored in the *35S::Pp3c21_7570* transgenic lines, although it is not as strong as in wild type (WT) plants (**Figure 2D**). These data suggest that *Pp3c21_7570* can partially complement the mutant phenotypes of *npr1-1*. Thus *Pp3c21_7570* is orthologous to Arabidopsis NPR1 and we named Pp3c21_7570 as PpNPR1.

The partial complementation of the Arabidopsis *npr1* mutant phenotypes by *PpNPR1* indicates that it functions as a positive regulator of SA-induced defense gene expression, suggesting that similar mechanisms are used to promote defense gene expression and pathogen resistance by SA in *P. patens*. Since there is only one NPR1-like protein in *P. patens*, it probably does not use NPR3/NPR4-like SA receptors to negatively regulate defense responses. As NPR3/NPR4 evolved from the same ancestor as NPR1 in higher plants, they probably diverged from NPR1 to take a different role in fine-tuning SA-induced defense responses. Whether *P. patens* has a simpler immune system which does not need negative regulators like NPR3/NPR4 or it uses an alternative approach to prevent auto-activation of defense responses in the absence of SA remains to be determined.

Bryophytes are remnants of early diverging lineages of embryophytes and occupy an ideal phylogenetic position for reconstruction of ancient evolutionary changes (Rensing et al., 2008). The identification of PpNPR1 as a NPR1-like protein provides a starting point for analyzing SA perception in the bryophyte *P. patens*. Future analysis of the potential SA-binding activity of PpNPR1 and characterization of the function of *PpNPR1* by knockout analysis will help us to better understand its roles in SA perception and defense against pathogens. In Arabidopsis, NPR1 interacts with TGA transcription factors and regulates defense gene expression through TGA2/TGA5/TGA6 (Zhang et al., 1999, 2003; Despres et al., 2000; Zhou et al., 2000). It is likely that PpNPR1 also interacts with TGA transcription factors in *P. patens*. It will be interesting to determine which TGA transcription factors in *P. patens* interact with PpNPR1 and analyze their roles in SA-induced defense gene expression.

## Author Contributions

YP performed the experiments and wrote the paper; TS performed the experiments; YZ designed the experiments and wrote the paper.

## Conflict of Interest Statement

The authors declare that the research was conducted in the absence of any commercial or financial relationships that could be construed as a potential conflict of interest.
